# Transforming individuals and institutions through competency-based faculty development

**DOI:** 10.3389/fmed.2025.1621376

**Published:** 2025-06-30

**Authors:** Mariah L. Robertson, Joseph Cofrancesco Jr, Rachel B. Levine

**Affiliations:** Johns Hopkins Medicine, Johns Hopkins University, Baltimore, MD, United States

**Keywords:** faculty development, competency-based education, medical education, academic promotion, health professions education

## Abstract

Faculty development (FD) is foundational to advancing the quality of health professions education and, by extension, the healthcare system. While FD has traditionally emphasized teaching, its scope now includes educational leadership, scholarship, and systems-level change. This Perspective argues for the adoption of competency-based FD frameworks to not only support individual educator growth but also catalyze institutional transformation. Aligning FD programming with established educator competencies fosters professional identity, enables targeted skill development, and legitimizes educational contributions in academic medicine. Drawing from global models—including frameworks from the United States, United Kingdom, the Netherlands, and Lebanon—we illustrate how institutions can adapt existing structures to their specific needs. We describe our experience at Johns Hopkins School of Medicine, where a locally tailored competency framework was developed to define six core educator domains and associated metrics. This initiative has driven significant changes in FD offerings, educator recognition, and promotion processes. Key lessons include the importance of making educator work visible, aligning FD with promotion pathways, delivering flexible programming, and engaging stakeholders early. Adapting frameworks to local contexts clarifies institutional values, supports customized faculty programming, and strengthens advocacy for educational excellence. Ultimately, competency-based FD serves as a strategic tool to enhance faculty satisfaction, retention, and institutional impact. We encourage institutions to adopt a systems-oriented approach that integrates shared standards with local relevance to elevate educational practice and culture.

## Introduction

Faculty development (FD), defined as activities designed to enhance the knowledge, skills, attitudes or behaviors of health professionals in their roles as educators, has long been recognized as a cornerstone of health professions education (HPE) (1, 2). Across the literature, FD encompasses building skills in teaching, but expands beyond that to include leadership, research, scholarship and larger scale organizational change (3) Health Professions Education (HPE) continues to evolve rapidly, and these shifts have introduced new tensions and demands on educators, such as compressed preclinical curricula, the implementation of competency-based medical education (CBME), increased emphasis on interprofessional education, and persistent concerns related to bias and disparities in education. In some instances, external regulatory agencies require institutions to demonstrate opportunities for faculty development in core topics (teaching skills, curriculum development, learner assessment, instructional methods, research) to ensure educational programs can achieve their stated learning outcomes. Faculty navigating this dynamic educational landscape need structured support to develop skills and to feel effective and fulfilled in their work. Institutions must recognize the strategic value of comprehensive FD initiatives, not only for faculty satisfaction and retention but also for academic advancement and long-term institutional success.

While the importance of FD is established, without deliberate integration, FD initiatives may fail to foster environments in which educators feel seen, supported, and empowered to succeed in their multifaceted roles. Using a competency framework can provide structure for thoughtful planning and evaluation of FD, however, faculty educator competencies are not comprehensively applied (3, 4). In addition to benefiting individual faculty, well-designed, competency-based FD initiatives can drive institutional transformation in support of educators and educational priorities (3). Competency frameworks for medical educators already exist. Building faculty development programming around these frameworks provides an opportunity to ensure consistency across settings and align efforts with shared professional standards (5–8).

This perspective piece argues that faculty development for health professions educators extends far beyond teaching instruction—it is integral to scholarly activity, leadership, and administrative success. We share lessons learned from our own experience adopting a competency-based framework to design and implement comprehensive FD initiatives focused on developing the knowledge, skills, attitudes and behaviors of our teaching faculty and also to support a shift in institutional support and recognition of educator faculty. We also share specific global models that highlight the breadth of ways that faculty development may take form, this is not meant to be an exhaustive review, but rather to give some varied examples for consideration. Our aim is to encourage readers to adopt an intentional, systems-oriented approach to FD that responds to institutional needs while leveraging shared language and standards to ensure quality and coherence within HPE.

## Faculty development as a catalyst for personal and institutional growth

Faculty development is essential not only for workforce development but also for cultivating and sustaining the professional identity of educators within academic health systems (9). By clearly articulating what educators do, faculty development initiatives provide a structured foundation for recognizing, supporting, and advancing educational roles across institutions. When aligned with a competency-based framework, FD programming can move beyond skills training to define and legitimize the work of teaching and facilitating learning; mentoring, coaching and advising; assessment and evaluation; educational leadership; curriculum and program development; and educational scholarship as vital, promotable forms of academic contribution (10).

Framing faculty development as a tool for advocacy and organizational change requires skilled leadership to advocate more effectively for resources and institutional recognition. Faculty development that includes a focus on educational leadership equips faculty with the tools to take on strategic roles within their institutions—roles that require not only pedagogical skill, but also the capacity to build programs, influence policy, and shape institutional (11). These educational leaders are then positioned as champions to negotiate for dedicated funding, protected time, and meaningful recognition of teaching excellence and innovation.

Moreover, faculty development can help to clarify and promote educational scholarship. By providing shared definitions and expectations for what constitutes scholarly work in education, FD initiatives support faculty in documenting their contributions and seeking promotion based on educational activities. This is particularly important in academic environments where research and clinical productivity are often more visibly valued than educational excellence (12). We considered these factors when developing our own FD programming discussed later.

## Global models of competency frameworks

Faculty development can take many forms. There is no one-size-fits-all approach to implementation. There is, however, a growing international consensus of core components of FD and we have much to learn from both shared experiences and local approaches (3). While some institutions may have the capacity to develop longitudinal courses or fellowships—structures that promote sustained transformation in teaching practice and foster communities of practice—others may rely on shorter interventions such as seminars, workshops, or coaching. All of these formats can meaningfully contribute to the growth and support of the educational workforce.

Applying a competency framework to faculty development design offers strategic scaffolding for such efforts (8, 13). As noted, beyond supporting knowledge acquisition and skill-building, these frameworks contribute to faculty professional identity formation, advocate for educator roles, and promote recognition and reward systems aligned with teaching innovation and excellence.

Although institutional contexts vary, a number of guiding frameworks can inform the design of tailored faculty development programs. In the United States, the Accreditation Council for Graduate Medical Education (ACGME) sponsored thought leaders to develop a comprehensive model of clinician educator milestones specifically designed for educators to use for self-assessment and self-directed learning. Training programs and other entities may use this framework to identify areas faculty development programming.

Similarly, in the United Kingdom, the Academy of Medical Educators (AoME) has developed *Professional Standards for Medical, Dental, and Veterinary Educators*, now in its fourth edition. These standards articulate five core values of medical educators and define five practice domains with associated competencies that support career progression and professional excellence along a developmental continuum (6).

The Netherlands Council of Deans of Medical Schools has taken a comparable approach by establishing a national task force to develop criteria for evaluating medical educator qualifications. They have developed domains across the micro, meso and meta levels. These domains provide a comprehensive framework for evaluating the qualifications of medical educators, ensuring that they possess the necessary competencies to contribute effectively at multiple levels within the educational system. This model can be modified to fit local settings and needs (6, 7).

## Practical guidance for local implementation

### The Beirut example

Daouk-Öyry et al. focused on identifying and developing a competency framework tailored for academic physicians in a teaching medical center in Beirut, Lebanon (14). They addressed the gap between the multifaceted roles of academic physicians and their education, emphasizing the need for a structured competency framework.

The study involved semi-structured interviews with 25 academic physicians to explore behaviors in teaching, clinical, research, and administrative roles. Through content analysis, they identified 16 competencies, categorized into five “Supporting Competencies” common to all roles and 11 “Function-Specific Competencies” unique to specific roles.

The framework shares similarities with existing competency-based models but includes unique elements tailored to their specific organizational context. It aims to bridge the gap between the skills taught to medical students and those required of academic physicians, with a future orientation and a focus on administrative skills.

### The Johns Hopkins example

At our institution, Johns Hopkins University School of Medicine, we have used a competency framework to build on established longstanding skills-development focused FD programs. This expanded scope promotes institutional change, specifically around the recognition, support, and promotion of educator faculty. Our work started with a problem common to many academic health centers with multiple missions; faculty whose primary career focus is education do not feel valued and report slower career advancement (15, 16). Several school-wide committees over the past 20 years had attempted to address this problem, producing sequential comprehensive reports recommending recognition of educators. Structural innovations followed including the creation of the Institute for Excellence in Education and comprehensive curricular reform in the medical school, but meaningful efforts to ensure the recognition and promotion of educators failed to gain traction (17). In 2019, a committee was formed to revisit this problem once again. This challenge had become particularly salient over the past decade as trainees and faculty have been seeking out programs to develop expertise in education including participation in medical education tracks in medical school and postgraduate training and advanced degrees in Health Professions Education. Faculty and trainees were increasingly choosing careers as educators and the lack of supportive institutional environments that recognized their contributions was more palpable. The issue had become one of faculty satisfaction and retention.

The Educator Competencies and Metrics Committee (ECMC) formed by the Executive Vice Dean in 2019, comprised a diverse group of faculty and staff, from across the institution representing the educational continuum. The committee unanimously agreed that using a competency framework to describe the activities of educators would provide broad structure for the design of FD programming. The committee also understood that this framework lent itself to developing clear and meaningful metrics for recognizing educational excellence and scholarship, thus addressing a primary goal of the committee: academic promotion for educators (12, 18).

After reviewing several educator competency frameworks as guides, we focused on describing what Johns Hopkins School of Medicine educators do in their daily work to develop a local framework that met our needs. The Johns Hopkins Educator Competencies and Metrics (ECM) framework includes 6 domains of educator work (Teaching and Facilitating Learning; Educational Leadership; Mentoring, Coaching and Advising; Program and Curriculum Development; Educational Scholarship; Assessment and Evaluation). Each domain includes subdomains with competencies. As our goal was also to ensure recognition and promotion of faculty educators, we also developed metrics for each domain which describe examples of educational activities and impact designed to align with local promotion criteria.

An example domain, sub-domain and associated competencies within our competency framework is the domain of *Educational Leadership*, under which an example sub-domain is *Reflection* and the associated competencies include:

- Solicits and incorporates ongoing feedback from all constituents.
- Demonstrates an awareness of how their behavior affects others.
- Seeks advice, feedback, or coaching from others in order to become a better leader.
- Helps team members succeed and grow into future leaders.

The work of this committee has yielded significant changes, including the development of a comprehensive set of faculty development offerings. These offerings are aligned with the key domains of educator work and address specific competencies within those domains. They are available in various formats to meet the needs of educational programs and individual faculty. The framework has also been used to create a set of institutional educational awards to more fully reflect and celebrate the work of educator faculty.

As a tool for advocacy, we have also addressed the promotion process by using the domains in promotion materials to highlight the impact of educational activities. This includes creating a common language in the CV and required promotion nomination letter and using the metrics to successfully argue for major changes to the promotion system. The institution is considering the creation of a separate educator promotion track using the work of the ECMC as a guide. Lastly, having detailed descriptions of educator roles has provided a stronger basis for advocacy around compensation for teaching and other educational work. Table 1 demonstrates the various stakeholders considered in the process and the benefits of using a competency framework for each.

**Table 1:** The benefits of a competency framework for Johns Hopkins University School of Medicine (JHUSOM) stakeholders.

JHUSOM stakeholder	Benefit/use of competency framework
Faculty	Professional development guide wherein a faculty member identifies domains of interest and pursues appropriate faculty development opportunities and training.
Mentors division/department directors	Provides targeted guidance on career pathway. Advises on career advancement and readiness for promotion.
Faculty development	Guides investments in faculty development resources. Focuses programming to ensure faculty are up-to-date with best practices in teaching, mentoring, coaching and advising, curriculum and program development and assessment and evaluation methods.
Educational leadership	Supports workforce development efforts through the definition of educator roles and job descriptions for recruitment.
Promotions committees	Supports consistent fair and transparent performance and promotion expectations.
Macro level – Accrediting bodies, learners, public, biomedical education	Quality assurance. Ensures the institution is in compliance with national requirements for faculty training. Guides improvements and innovations in biomedical education.

One key lesson learned from the evolution of our faculty development work is the importance of making educators’ work visible. The use of a competency framework not only guides individual growth and curriculum design but also creates a common language for evaluating and advocating for the role of educators in academic medicine (9, 12).

There were many lessons learned in our institutional faculty development efforts. These include:

- *Faculty development is foundational*: Every domain of educator work should intentionally include faculty development to support educator growth. This ensures capacity building in key areas of educational expertise and support for educational programs. Having a detailed set of competencies allows for planning and advocacy for resources for faculty development and education in general.
- *Flexibility enhances engagement*: We have learned to adapt existing faculty development programming to be provided in shorter, more consumable and easily accessible formats to allow for greater flexibility while maintaining alignment with core educator competencies. In addition, training a cadre of local faculty development experts at our large institution has been critical to providing specialty specific FD offerings. Using a competency framework allows faculty to “specialize” in areas of interest and develop expertise.
- *Institutional advocacy matters*: Supporting educators through local recognition efforts—such as educator awards, promotion pathways, and resource allocation—can drive meaningful cultural change and elevate the value of education. A competency framework makes the work of educators visible and thus creates a platform from which to advocate for educators. During the implementation process regular communication with all institutional stakeholders is critical. A competency framework can also help to match educator activities to institutional priorities, for example around accreditation of educational programs.

## Discussion

### Key considerations for building successful, context-specific faculty development

When considering how to build or enhance a faculty development program, one critical starting point is identifying and implementing a competency framework. Such frameworks provide structure and clarity by defining the core knowledge, skills, and attitudes educators need to thrive in their roles. For anyone undertaking this work, you might ask the first question: *Do I want to create a new framework, or can I adapt an existing one tailored to my institution’s needs?* Adopting an established framework can offer clear benefits, including alignment with national or global standards, reduced development time, and access to accompanying tools or resources. However, local adaptation is often necessary to ensure relevance, and this process can be just as valuable—prompting institutions to clarify what they value in educational work, much like was done in the Beirut and Johns Hopkins examples. It is also important to consider the purpose of using a framework with questions such as: *Will it be used to guide programming design, support faculty self-assessment and growth? Will it align with promotion criteria to help make educational contributions more visible and measurable?* Mapping program content to a competency framework can make faculty development more transparent, outcomes-focused, and better aligned with institutional goals. Additionally, thinking about key stakeholders—such as department chairs, education leaders, faculty affairs offices, and educators themselves—can help clarify how different groups might benefit and to ensure buy-in during the process. For example, faculty may gain clearer expectations and support for their development, while leadership can use the framework to advocate for resources, define excellence in education, or even build pathways for educator promotion. Ultimately, the value of a competency framework lies not just in its content, but in how thoughtfully it is selected, adapted, and applied within a local context.

### Summary

Effective faculty development programs are critical to advocating for faculty educators and driving institutional change. In our own work, we have identified several key lessons learned that highlight the importance of designing flexible and responsive programming that can adapt to evolving faculty and institutional needs (Table 2). Meeting faculty where they are happens when we recognize the many stages of professional identity formation and growth. Building both individual and institutional capacity in teaching, leadership, and scholarship is essential to sustaining change efforts. Engaging a diverse range of stakeholders early fosters shared ownership and aligns goals across the institution. The use of competency frameworks helps surface, legitimize, and elevate the educational contributions of faculty, while frequent and broad communication helps socialize ideas and galvanize momentum for change. Targeted assessments ensure that programming remains relevant and responsive to faculty needs. Finally, involving faculty developers directly in the planning and implementation of curricular and institutional initiatives strengthens the efforts and ensures success.

**Table 2:** Lessons learned and best practices based in the literature and JHUSOM experience.

Theme	Lesson learned/best practice
Adaptability	Design flexible and responsive programming that can evolve with institutional and faculty needs.
Developmental orientation	Meet faculty where they are by recognizing their varying stages of professional identity and growth.
Capacity building	Build individual and institutional capacity in teaching, leadership, and scholarship.
Stakeholder engagement	Engage diverse stakeholders early to align goals and foster shared ownership.
Visibility of educational work	Use competency frameworks to surface and legitimize the educational contributions of faculty.
Strategic communication	Communicate frequently and broadly to socialize ideas and galvanize institutional change.
Needs assessment	Conduct targeted assessments to inform programming and ensure relevance to faculty needs.
Inclusive innovation	Involve faculty developers in the planning and implementation of curricular and institutional change.
Change advocacy	Leverage faculty development as a platform for advancing educational equity and institutional reform.
Faculty well-being	Promote connection and satisfaction through community-building and shared purpose.
Recognition and value	Use FD to affirm the value of educational roles and support pathways for recognition.
Leadership pathways	Position faculty development as a conduit for leadership opportunities and promotion.
Program evaluation	Plan for program evaluation to demonstrate meaningful outcomes and advocate for ongoing resources and support.

References: (7, 11, 12, 18–23).

## Data Availability

The original contributions presented in the study are included in the article/supplementary material, further inquiries can be directed to the corresponding author.
